# Dynamics of Contrast Decrement and Increment Responses in Human Visual Cortex

**DOI:** 10.1167/tvst.9.10.6

**Published:** 2020-09-04

**Authors:** Anthony M. Norcia, Alexandra Yakovleva, Bethany Hung, Jeffrey L. Goldberg

**Affiliations:** 1 Department of Psychology, Stanford University, Stanford, CA, USA; 2 Department of Ophthalmology, Stanford University, Stanford, CA, USA; 3 Johns Hopkins University School of Medicine, Baltimore, MD, USA

**Keywords:** visual evoked potentials, OFF pathway, ON pathway, luminance contrast, latency

## Abstract

**Purpose:**

The goal of the present experiments was to determine whether electrophysiologic response properties of the ON and OFF visual pathways observed in animal experimental models can be observed in humans.

**Methods:**

Steady-state visual evoked potentials (SSVEPs) were recorded in response to equivalent magnitude contrast increments and decrements presented within a probe-on-pedestal Westheimer sensitization paradigm. The probes were modulated with sawtooth temporal waveforms at a temporal frequency of 3 or 2.73 Hz. SSVEP response waveforms and response spectra for incremental and decremental stimuli were analyzed as a function of stimulus size and visual field location in 67 healthy adult participants.

**Results:**

SSVEPs recorded at the scalp differ between contrast decrements and increments of equal Weber contrast: SSVEP responses were larger in amplitude and shorter in latency for contrast decrements than for contrast increments. Both increment and decrement responses were larger for displays that were scaled for cortical magnification.

**Conclusions:**

In a fashion that parallels results from the early visual system of cats and monkeys, two key properties of ON versus OFF pathways found in single-unit recordings are recapitulated at the population level of activity that can be observed with scalp electrodes, allowing differential assessment of ON and OFF pathway activity in human.

**Translational Relevance:**

As data from preclinical models of visual pathway dysfunction point to differential damage to subtypes of retinal ganglion cells, this approach may be useful in future work on disease detection and treatment monitoring.

## Introduction

In the vertebrate retina, two parallel pathways diverge from the first synapse between the photoreceptors and bipolar cells: one signaling luminance increments (ON) and the other signaling luminance decrements (OFF).^1^ These dual pathways flow from bipolar to retinal ganglion cells (RGCs), whose dendrites stratify in different retinal sublaminae,^2^^,^^3^ and they remain segregated in the lateral geniculate nucleus.^4^^,^^5^ Visual cortex also retains a degree of pathway segregation.^6^^–^^11^

The spatial properties of the two pathways have been extensively documented primarily in nonhuman primate and cat. The receptive fields of OFF RGCs are smaller than those of their ON counterparts,^12^^–^^15^ as are their dendritic arbors.^14^ OFF RGCs are also more numerous,^16^ and the cortical tiling of OFF inputs is more focal than ON tiling.^11^^,^^17^ OFF responses dominate ON responses in cortex,^18^^–^^20^ and the contrast response function also differs for increments and decrements.^21^ Together, these factors may explain the reported higher spatial resolution for darks than lights.^22^ Analyses of natural image statistics suggest the adaptive value of this asymmetry: negative (dark) contrasts are more prevalent than positive (light) contrasts in natural scenes,^16^^,^^23^^,^^24^ and efficient coding of natural images would thus benefit from the observed asymmetry.

Differences in the dynamics of the two pathways have been less studied. An early in vitro study in primate RGCs found faster responses in ON versus OFF cells.^12^ This work was cited as a possible basis for an apparent motion illusion where it was estimated that brights were processed ∼3 ms faster than darks.^25^ More recent in vivo work in cat has found that OFF cells in the Lateral Geniculate Nucleus (LGN)^26^ and in visual cortex^27^ respond more quickly than ON-dominated cells. This latter physiologic ON/OFF asymmetry is consistent with other human psychophysical work that has found that darks are processed faster than lights.^28^ Spatial and temporal factors appear to be coupled in that small, brief stimuli drive stronger OFF than ON responses in cat visual cortex, while the converse is true for large, longer duration targets.^29^

Relatively little work has been done to study or characterize ON versus OFF pathways in humans. Visual evoked potentials (VEPs) provide a possible means of measuring the response properties of the two pathways noninvasively. Several VEP studies have compared responses to contrast increments and decrements^22^^,^^30^^–^^33^ on the assumption that luminance decrements are preferentially processed by the OFF pathway and vice versa.^34^ Here we build on this prior research by presenting sawtooth increments and decrements using a spatially optimized stimulation array and large study samples. The use of sawtooth stimulation was motivated by prior psychophysical work suggesting that it selectively stimulates separate perceptual channels^35^^,^^36^ and that fast decremental sawtooth stimulation elicits lower contrast thresholds than fast incremental stimulation.^37^ Sawtooth stimulation also activates ON versus OFF center retinal ganglion cells in a differential fashion.^34^ When using sawtooth stimulation, our measurements indicate that steady-state visual evoked potentials (SSVEPs) to decrements are typically larger and faster than those to corresponding increments.

## Methods

### Participants

A total of 108 adults between the ages of 18 and 80 years participated. All participants had visual acuity of 20/25 or better in each eye on the Bailey-Lovie constant LogMAR chart and a stereoacuity of 50 arcsec or better on the RandDot test. The experiments proceeded after written informed consent was obtained using a consent form approved by the Institutional Review Board of Stanford University. The research conformed to the tenets of the Declaration of Helsinki for use of human subjects.

### Visual Stimuli

We report the results of five experiments. The first experiment was designed to optimize the stimulus spatial parameters, the second was designed to obtain full-field data from younger participants with a modified array and an attentional control, the third to compare older and younger participant responses to increments and decrements, the fourth to compare upper and lower hemifield responses, and the fifth to compare contrast polarity effects under equal Michelson contrast conditions. Demographic and procedural details that varied over each experiment are provided in the Table. Common features across experiments are described below.

**Table.** Demographics and a Subset of Stimulation Parameters for Each Experiment

Comparison	Experiment	No.	No. of Females	Age, y	Viewing Eye	Core Trial Duration	Task	Randomization
Size	1	11	6	25.0	Binocular	12	Fixate	Trial level
Polarity	2	14	8	19.3	LE, RE	9.9	Letter	Trial level, blocked by eye
Age (younger)	3	12	4	19.2^a^	LE, RE	11.1	Letter	Trial level, blocked by eye
Age (older)	3	19	6	57.1	LE, RE	7.7	Letter	Trial level, blocked by eye
Hemifield	4	28	11	20.0	Dominant	11.1	Fixate	Trial level
Control (Low)	5	12	4	19.2^a^	LE, RE	11.1	Letter	Trial level, blocked by eye
Control (High)	5	24	9	19.5	LE, RE	11.1	Letter	Trial level, blocked by eye
	Grand totals	108	44					

LE, left eye; RE, right eye.

a Same participants.

**Figure 1.**
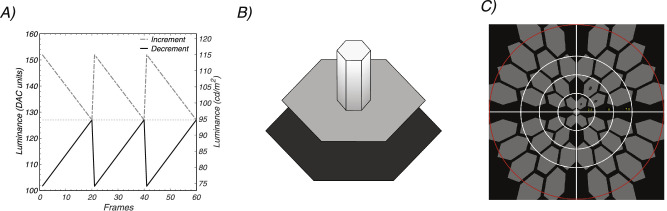
(A) Probe waveforms. Incremental (*gray*) and decremental (*black*) sawtooth waveforms designed to favor ON versus OFF pathway responses, respectively. A stimulus frequency of 3 Hz is illustrated. Frame rate was 60 Hz. *Dashed line* indicates pedestal luminance. The *left* ordinate plots the digital to analog converter values (DAC) used. (B) Probe on pedestal display element. The sawtooth-modulated probes (*small white hexagon*) were presented on a *mid-gray* pedestal (*medium size hexagon*). An incremental pedestal is illustrated. The probe was 20% the size of the pedestal. The pedestal was surrounded by a *black* background region (*largest hexagon*). Weber contrast was 20% for both increments and decrements. (C) Scaled stimulus array. The visual field was tiled with a set of probe/pedestal elements. The size of the elements was scaled over eccentricity according to the cortical magnification factor to optimize responses from the periphery. Typical field size was ∼12 degrees in radius (rings indicate 2.5-degree eccentricity radii from central fixation).

We used periodic decremental and incremental sawtooth stimulation to elicit SSVEPs biased to OFF versus ON pathways, respectively.^34^^,^^37^ Decremental stimuli were defined as those in which the fast phase of the sawtooth decreases in luminance and the slow ramp phase increases (Fig. 1A, gray curve), with incremental stimuli being the opposite (Fig. 1A, black curve). The stimulus frequency was 3 Hz for the first experiment and 2.73 Hz for rest of the experiments.

The spatial structure of the stimulus was based on the Westheimer sensitization paradigm.^38^^,^^39^ In our version of the paradigm, a small probe that was modulated according to either the decremental or incremental sawtooth profile was presented on a larger static background pedestal (Fig. 1B). Prior psychophysical work has demonstrated that decrements produce sensitization effects that are comparable to those for traditionally used increments.^40^ In all of the experiments, the probes were presented at a fixed modulation depth of +20% (increments) and –20% (decrements) Weber contrast, calculated as the change in probe luminance divided by the pedestal luminance. Increments and decrements had opposite signs but equal values under this definition. The dashed line in Figure 1A indicates the pedestal luminance.

Stimuli were presented on SONY (Sony Corporation, Tokyo, Japan) PVM-2541 monitors (1920 × 1080 pixels). Participants’ electroencephalogram (EEG) data were collected at two facilities that used identical hardware. For both locations, display and EEG pipeline delays were measured with a photocell and have been corrected. Most of the measurements reported here were made with younger participants at a pedestal luminance of 94.5 cd/m^2^. Data from the older participants were collected at a clinical research facility. These observers were serving as control observers for a parallel study of patients with glaucoma. Due to patient comfort considerations, the luminance of the display was lowered to 47.3 cd/m^2^ and the trial duration was shortened. Viewing was binocular in experiment 1 and monocular in the remaining experiments. When viewing was monocular, the other eye was covered with an opaque patch. Viewing distance was 70 cm. Stimulus conditions were presented in random order, and an intertrial interval of 3000 ms was used.

### Experiment 1: Visual Stimuli and Participants

**Figure 2.**
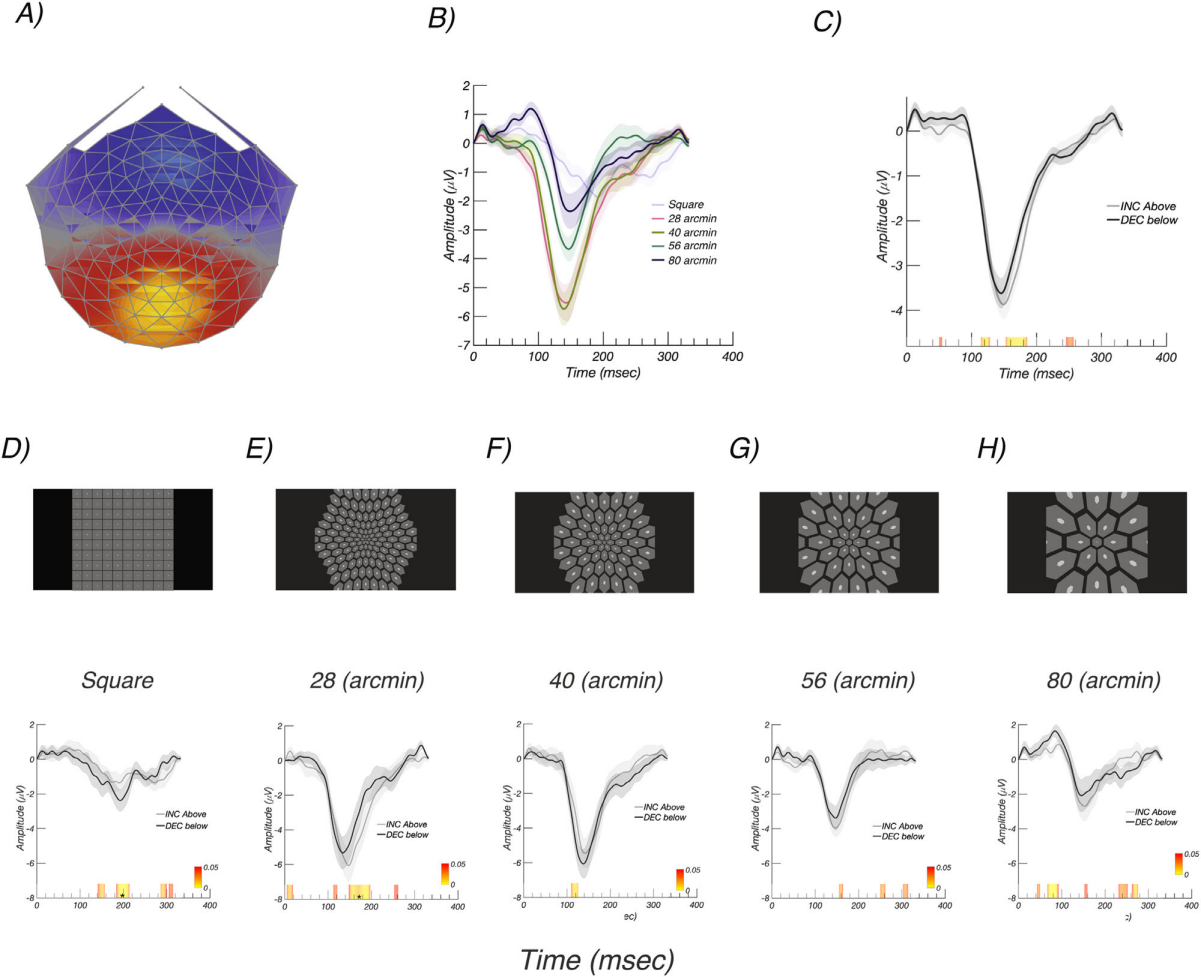
Experiment 1: Array scale tuning. (A) Response topography of the RC1 component learned over all array sizes. Response is maximal over Oz. (B) Responses to different array sizes pooled over contrast polarity (see legend for condition labels). Responses are largest for the 40-arcmin array. (C) Responses to increments/ON (*gray*) and decrements/OFF (*black*) pooled over array size. Responses to increments and decrements are comparable. (D–H) *Top panels* present schematic illustrations of the stimulus arrays. (D–H) *Bottom* *panels* show corresponding RC1 evoked responses with increments/ON in *gray* and decrements/OFF in *black*. See text for details. *Color bar* indicates *P* values for the difference between conditions, starting at *P* < 0.05 in *red*. *Asterisks* indicate runs that pass the run correction criterion at *P* < 0.05.

This experiment compared incremental and decremental responses for five stimulus configurations (see Figs. 2D–H). One stimulus configuration (Fig. 2D) comprised a rectangular array of 100 elements with the probe being 10.5 by 10.5 arcmin and the pedestal 136 by 136 arcmin. The entire array subtended 23.8 by 23.8 degrees when viewed at 70 cm. Four additional configurations (see Figs. 2E–H) comprised hexagonal arrays of probe-on-pedestal elements. These arrays also covered 23.8 by 23.8 deg and were viewed at 70 cm. The base hexagonal element in the center of the visual field varied between 28 and 80 arcmin for the pedestal and between 5.6 and 16 arcmin for the corresponding probes, with the pedestal always five times larger in linear extent than the probe. The probe, pedestal, and background luminance values were the same as for the rectangular array described above.

The size of the array elements was scaled based on prior estimates of the cortical magnification factor.^41^ First, we generated a hexagonal grid, with the spacing between elements being defined by the element base size (in pixels) and the total number of elements as limited by the screen dimensions. To avoid potential overlapping issues and improve visual perception, the grid was calculated for slightly larger hexagons by a linear factor of 2/√3.

The hexagonal centers h_0_ were used for both probe and pedestal generation. Next, for each hexagon of size h^i^ centered at $(x_{0}^{i}\;,{\;y}_{0}^{i}),$ we computed vertex coordinates (x^i^,y^i^) in the Cartesian coordinate system.

$$\begin{array}{c} \alpha =\left[0,\;\frac{\pi }{3},\;\frac{2\pi }{3},\;\pi ,\;\frac{4\pi }{3},\;\frac{5\pi }{3}\right] \end{array}$$

$$\begin{array}{c} x^{i}=h^{i}\;*\;\sin\alpha +x_{0}^{i} \end{array}$$

$$\begin{array}{c} y^{i}=h^{i}\;*\;\cos\alpha +y_{0}^{i} \end{array}$$

These vertices were then converted to polar coordinates [θ^i^,  ρ^i^] using MATLAB's cart2pol function:

$$\begin{array}{c} \left[\theta^{i},\;\rho^{i}\right]=\mathrm{cart}2\mathrm{pol}\left(x^{i},\;y^{i}\right) \end{array}$$

followed by a k-factor magnification (k = 0.7 matching cortical magnification factor) applied to radial coordinates using the following equation in the polar coordinate system:

$$\begin{array}{c} s^{i}=\;\rho^{i}\left(1+\;\frac{\rho^{i}}{k}\right) \end{array}$$

We then converted unchanged angular and magnified radial coordinates [θ^i^, s^i^] back to Cartesian coordinates using the pol2cart function:

$$\begin{array}{c} \left[{\tilde{x}}^{i},{\tilde{y}}^{i}\right]=\mathrm{pol}2\mathrm{cart}\left(\theta^{i},\;s^{i}\right) \end{array}$$

Next, we marked regions inside the magnified polygonal shapes $\left[{\tilde{x}}^{i},{\tilde{y}}^{i}\right]$ using the poly2mask function, with outside pixel values being set at 0 and inside values set at 1. The resulting quarter-quadrant mask was mirror-reflected twice to fill the screen. To finish the process, we removed elements with centers on the x- or y-axis. We repeated this process for the pedestals and probes separately and summed the resulting images.

This experiment was conducted with 11 participants (six females, mean age 25 years). The experiment comprised 10 conditions (five spatial layouts and two contrast signs). Nine trials lasting 12 seconds each were collected per condition, and the order of presentation was randomized at the trial level.

### Experiment 2: Visual Stimuli and Participants

This experiment was designed to collect data with a display that eliminated elements that straddled the horizontal and vertical meridian (see Fig. 1C). This experiment used an 8-arcmin probe/40-arcmin pedestal display and was conducted with a group of 14 younger participants (eight females, mean age 19.3 years) at a pedestal luminance of 94.5 cd/m^2^. The entire array subtended 23.8 by 23.8 degrees when viewed at 70 cm. The participants performed a concurrent letter discrimination task presented with the central hexagon to control fixation and attention. Viewing was monocular, and each eye was tested. Trials lasted 9.9 seconds, and 15 trials were recorded in each of the four stimulus conditions (left eye (LE), right eye (RE), increments/decrements).

### Experiment 3: Visual Stimuli and Participants

This experiment compared responses to younger and older participants using the display array shown in Figure 1C. This experiment used an 8-arcmin probe/40-arcmin pedestal display and was conducted with a group of 12 younger (four females, mean age 19.2 years) and group of 19 older (six females, mean age 57.1 years) participants. Both younger and older participants stimulus sets contained four conditions: increments and decrements in each eye, with increments and decrements being presented in random order within an eye. Younger participants were presented with 11.1-second trials whereas older adults were presented with shortened 7.7-second trials, total of 12 trials, for better viewing comfort. In the statistical analysis, each eye was treated as an independent sample.

### Experiment 4: Visual Stimuli and Participants

This experiment was designed to measure the relative amplitudes of the response in the upper and lower visual fields and to test for field cancellation effects that may have been present in the full-field recordings. This experiment used an 8-arcmin probe/40-arcmin pedestal display and was conducted with 28 participants (11 females, mean age 20 years). Eighteen trials lasting 11.1 seconds were presented in random order for both increments and decrements, with each presented in both the upper and lower visual field (four conditions). The entire array subtended 23.8 by 23.8 degrees when viewed at 70 cm, but only half of the probes were presented. One participant took part in experiments 1, 2, and 4, and the rest of the participants were unique to each experiment.

### Experiment 5: Visual Stimuli and Participants

The increment above the pedestal and decrement below the pedestal sawtooth stimuli used in the previous experiments had the same Weber contrast but slightly different Michelson contrast, with decrements having about 20% more relative contrast. This experiment used sawtooth stimulation for increments and decrements that was symmetric about the pedestal, equating the Michelson contrasts and also retaining the standard 20% Weber contrast as used in the previous experiments. The experiment was conducted in younger participants (mean age 19.4 years, N = 36 total participants, 11 females) at two pedestal luminances, 94.5 cd/m^2^ and 47.3 cd/m^2^ with a probe size of 8 arcmin. Fifteen 11.1-second trials were run. The entire array subtended 23.8 by 23.8 degrees when viewed at 70 cm.

### SSVEP Recording

The EEG was recorded over 128 channels using Hydrocell SensorNets and NetStation 5.2 software (Electrical Geodesics, Eugene, OR). Prior to recording, individual electrodes were adjusted so that the impedance values were lower than 60 kΩ.

### Artifact Rejection and EEG Filtering

The raw EEG was amplified (gain = 1000 at 24-bit resolution) and digitally filtered with a zero-phase 0.3- to 50-Hz bandpass filter. The data were then processed using in-lab software written in Objective C. The artifact rejection procedure first detected and then substituted consistently noisy individual channels. The noisy channels were substituted with the average signals of the six nearest electrodes surrounding the noisy electrode. After this, the EEG was re-referenced to the common average of all electrodes. Second, in an effort to reject data recorded during coordinated muscle movements or blinks, 1-second-long epochs were excluded for all electrodes if signals of more than 5% (7 out of 128) of the electrodes exceeded a set threshold amplitude (60–520 µV; median, 100 µV) sometime during the epoch. Third, the first and last 1 second (experiment 1) and the first and last 1.1 seconds for experiments 2 to 5 were excluded from the data analysis. Finally, 1-second epochs from individual electrodes were excluded if more than 10% of the epoch samples exceeded ±30 µV.

### Reliable Components Analysis

Reliable components analysis (RCA) was used to reduce the dimensionality of the 128-channel recordings to a small number of components as described previously.^42^ Each RCA component comprises a scalp topography and a response time course or spectrum. RCA components were derived through an eigenvalue decomposition that maximized the trial-by-trial covariance matrix. This criterion reflects the fundamental assumption that the stimulus-driven evoked response is highly similar over repeated presentations of the same stimulus. RCA results in an improved signal-to-noise ratio and provides a data-driven method for selecting the recording channels whose data are to be analyzed. RCA was computed in the time domain using epochs that corresponded to the cycle length of the stimulus, which was 333 ms in experiment 1 and 366 ms for experiments 2 and 3. RCA in the frequency domain was computed on the basis of the complex values of the first four harmonics of the stimulus frequency from each second of the data record. We report data from the first RCA component as it reflects the responses that are most consistent on a trial-by-trial basis.

### Permutation Testing for Time-Domain Waveforms

Permutation-based testing was used to identify significant differences in response amplitude within groups. The hypothesis tested was whether there was a difference between the ON/increment and OFF/decrement responses (e.g., the null hypothesis was that the paired difference potential was zero). To create the empirical sampling distribution for this hypothesis, instances of the group mean difference potential were generated by first randomly permuting the sign of the ON/OFF difference potential for each participant and then averaging them.

For each within-group permutation test, 5000 synthetic data sets were created, and *t*-scores were computed using paired *t*-tests (MATLAB's **ttest** function). Continuous significant *t*-score durations were computed and added to a null distribution. We then computed *t*-scores and *P* values for the original ground-truth data set and rejected the null hypothesis for intervals that were longer than 95th percentile of the null distribution.

### Delay Estimation in the Frequency Domain

To quantify the magnitude of the timing difference between increments and decrements, we derived apparent latencies in the frequency domain by plotting response phase as a function of response frequency.^43^^,^^44^ Delay was estimated as d = 1/360 × δϕ/δf, where δϕ is the change in phase over the frequency range in degrees and δf is the change in frequency in Hz.

Fitting was done using a linear regression method, where the slope and intercept were obtained with MATLAB's **polyfit** function. Error values were calculated as the standard error of the estimate:

$$\begin{array}{c} \sigma =\sqrt{\frac{\sum_{i=1}^{N}{\left(y_{i}-{\hat{y}}_{i}\right)}^{2}}{N-2}} \end{array}$$

where *y*, $\hat{y}$ are observed and estimated phase-angle values and *N* is the number of observations, which is 4.

## Results

### Experiment 1: Scaling the Stimulus Array to Maximize Response Amplitude

The SSVEP stimuli in this experiment comprised multiple small elements because our ultimate goal is to probe ON versus OFF pathway responses in different parts of the visual field. Prior work on SSVEP responses to incremental versus decremental stimuli has used rectilinear arrays of probes.^22^^,^^30^^–^^33^ He we sought to determine what the best spatial scaling for the entire array would be to elicit the largest SSVEP. This scale would presumably be the one that drives the largest number of cells at each eccentricity. Because of cortical magnification, the probe and pedestal in the fovea should be smaller than the probes and pedestals in the periphery. To determine whether scaling the SSVEP test/pedestal stimulus for retinal eccentricity results in a larger SSVEP than an unscaled stimulus, we compared responses generated by several cortically scaled arrays to those from an unscaled array similar to one used in previous experiments on incremental and decremental SSVEP responses.^22^^,^^30^^–^^33^ Each of the scaled arrays used the same magnification factor. After some preliminary testing, we settled on a range of base element sizes for the central-most hexagon that spanned a factor of 4 in half-octave steps.

The scalp topography of the dominant evoked response component, RC1, was focused over the occipital pole, as can be seen in Figure 2A. The RC1 weights were learned over the data collected from the five stimulus arrays, which are shown schematically in Figures 2D–H, top.

We observed that the scaling of the array has a large effect on response amplitude. Responses for the different arrays, averaged over polarity, are shown in Figure 2B. The amplitudes evoked by the rectangular array (dark green trace) are smaller than any of the responses measured with scaled arrays. This is despite there being more probes in the rectangular array (100) than in three of four of the scaled arrays (125, 59, 35, and 19, respectively). The largest responses measured were for a central pedestal size of 40 arcmin (8-arcmin probe size; gray trace), where the responses were a factor of approximately 3 larger than for the rectangular array. Responses as a function of contrast polarity, averaged over array type, are shown in Figure 2C. Here the responses are of similar amplitude.

Time averages for each array are shown separately for increments (gray) and decrements (black) in the lower panels of Figures 2D–H. For each of the scaled arrays, the latency of the negative peak is slightly shorter for decrements than for increments. Around the time of maximum response amplitude (∼150 ms), responses to decrements are measurably larger than for increments for the square and 40-arcmin arrays. Given that the 40-arcmin scaled array evoked the largest response overall, this display scaling was used for the remaining experiments.

### Experiment 2: Full-Field Responses with Modified Array

In this experiment, we collected monocular data for incremental and decremental stimuli in a group of 14 younger participants (mean age 19.3 years). The 40-arcmin array of experiment 1 was modified so as to eliminate elements along the horizontal and vertical meridians (see Fig. 1C), as we wished to also study hemifield responses where cancellation effects could occur. Pedestal luminance was 94.5 cd/m^2^ and a Weber contrast of 20% was used for both decrements and increments.

**Figure 3.**
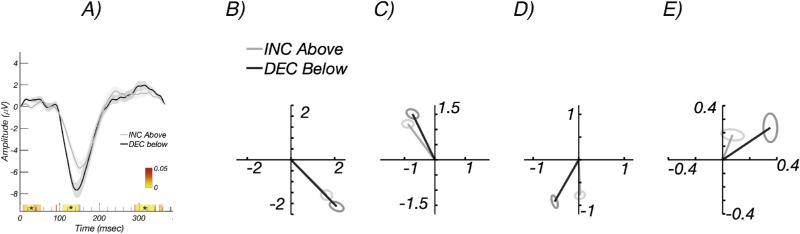
Experiment 2: Estimation of delay in the frequency domain. (A) Time-domain waveform for ON/increment (*gray*) and OFF/decrement (*black*) responses in the younger participant group. (B–E) Nyquist diagrams for 1F, 2F, 3F, and 4F, respectively, showing magnitude and phase of the evoked response for ON/increments (*gray*) and OFF/decrements (*black*). Responses to decrements are larger than for increments and are phase advanced relative to responses to increments. Phase origin is at 3 o'clock; increasing delay is in the counterclockwise direction. *Color bar* in (A) indicates *P* values for the difference between groups or conditions, with starting at *P* < 0.05 in *red*. *Asterisks* indicate runs that pass the run correction criterion at *P* < 0.05. *Error ellipses* are ±1 SEM.

Figure 3A shows increment versus decrement waveforms, with decrements in black and increments in gray. Responses to decrements and increments both show a prominent negative peak at around 140 ms, where the response is larger for decrements versus increments. It is also apparent that the peak of the response for decrements occurs slightly earlier. The colored band on the x-axis indicates the significance level of the difference between conditions

### Frequency Domain Estimate of Delay

**Figure 4.**
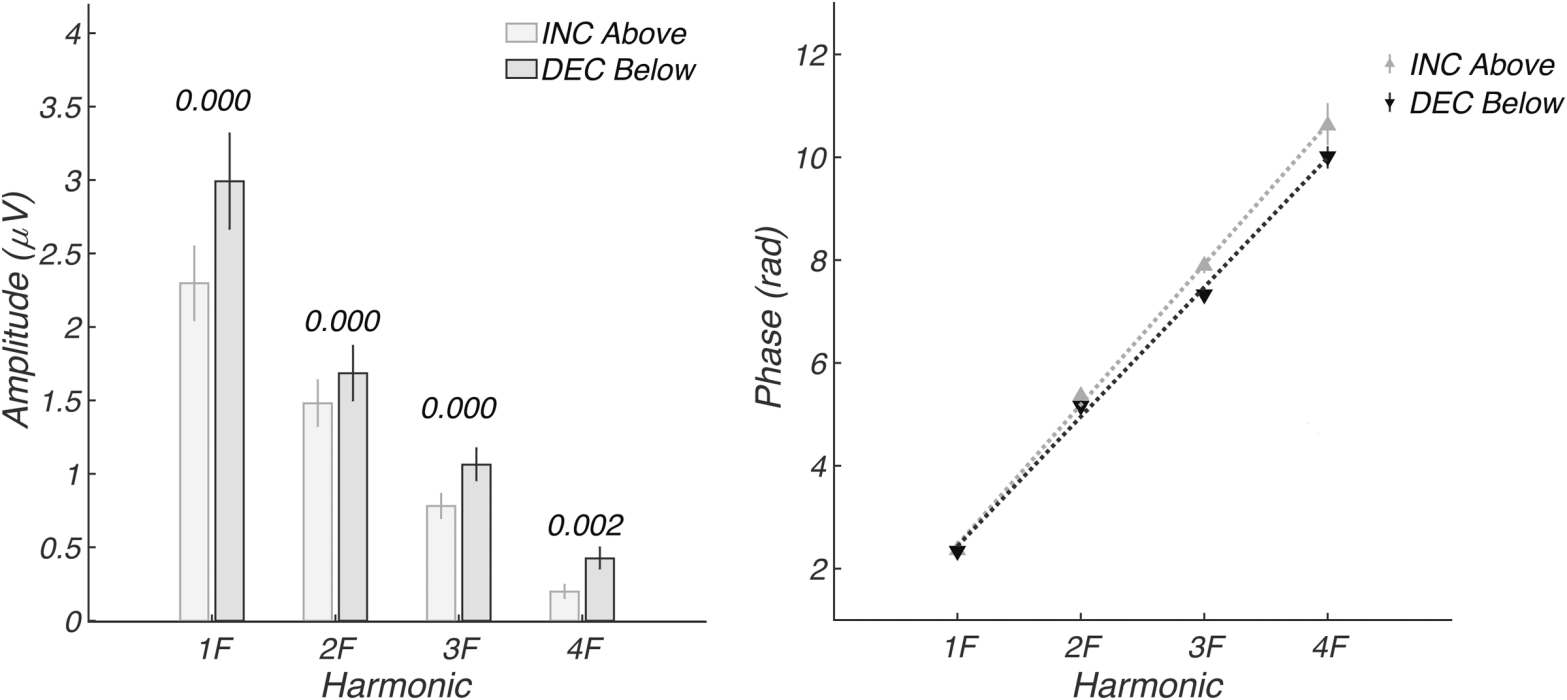
Experiment 2: Response amplitude and delay estimation in frequency domain. (A) Amplitudes at the first four harmonics of the stimulus frequency (1F, 2F, 3F, 4F) for the time-domain data shown in Figure 3. Values above the *bars* indicate the significance level of the within-participant difference. (B) Response phase in radians as a function of response harmonic. A shallower slope indicates less delay. The estimated delays for increment/ON and decrement/OFF responses are 159.2 ± 1.8 ms and 146.8 ± 2.4 ms, respectively. *Error bars* are ±1 SEM.

Because the evoked response was generated through a periodic stimulation paradigm, the response comprises a SSVEP. Estimating onset delay in the time domain in a periodic design is potentially confounded with wraparound effects, so we measured relative delay in the frequency domain. When considered in the frequency domain, the evoked response we measure consists of a series of narrow-band responses at exact integer multiples of the stimulus frequency. Figures 3B–E show the frequency domain representation of the same data over the first four harmonics of the 2.73-Hz stimulus frequency. The Nyquist diagrams for each harmonic plot the real coefficient magnitude on the x-axis and the imaginary coefficient magnitude on the y-axis. The response amplitude corresponds to the vector length, and the phase (delay) of the response is represented as the polar angle, with 0 delay being plotted at 3 o'clock. From these frequency domain measurements, we can see that the decrement responses are larger (longer vectors) and occur closer to the phase origin at 3 o'clock, consistent with them being less delayed with respect to the stimulus. The larger response to decrements can also be seen in Figure 4A, where response amplitudes are plotted as a function of harmonic number for the first four harmonics of the stimulus frequency. Responses to decrements/OFF are plotted in black and increments/ON in gray.

The response phases are plotted as a function of harmonic number in Figure 4B. Response phase is a linear function of harmonic number for both decrements (black) and increments (gray). This is consistent with the different response harmonics having been subjected to a fixed “group” delay between the stimulus and cortex. The slope of the phase function can thus be used to estimate a delay for the SSVEP in a way that accounts for the periodic nature of the stimulation and that does not require estimating when the response leaves baseline as it does in the time domain. The estimated delays for increment/ON and decrement/OFF responses are 159.2 ± 1.8 ms and 146.8 ± 2.4 ms, respectively, a difference of ∼13 ms.

### Experiment 3: Younger versus Older Participants

In this experiment, we collected monocular data for incremental and decremental stimuli in a group of 12 younger (mean age 19.2 years) and 19 older (mean age 57.1 years) adult participants. RCA was performed separately for the two age groups, combining data across eyes within an age group with the components learned jointly on the two stimulus polarities within an age group.

**Figure 5.**
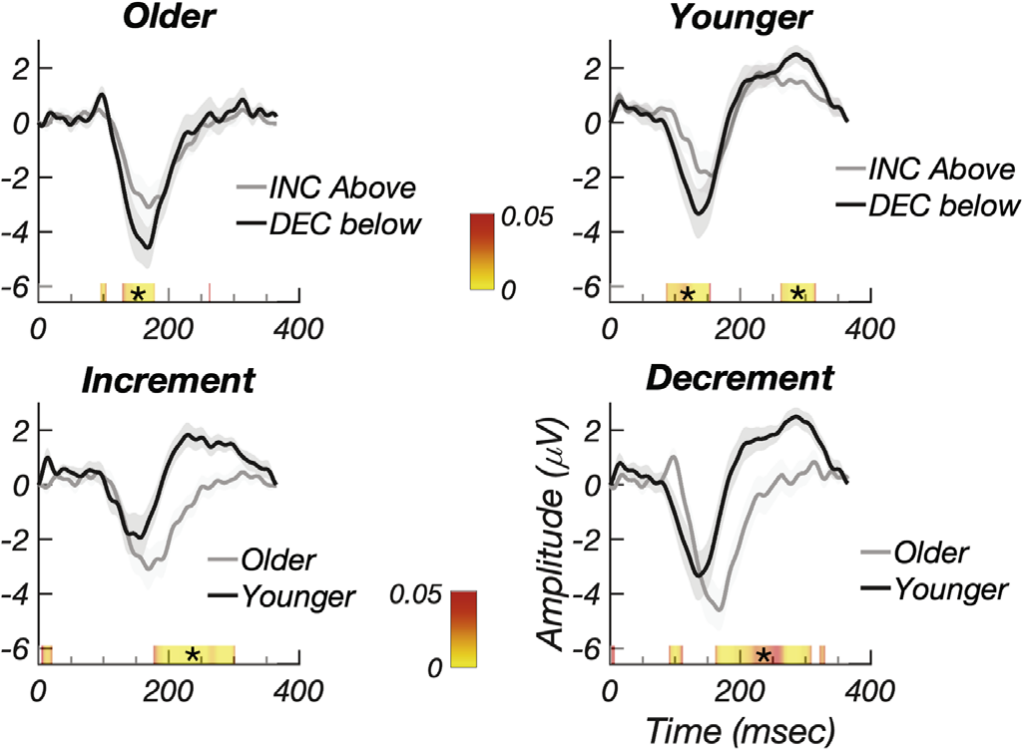
Experiment 3: (*Top left*) Increment/ON (*gray*) versus decrement/OFF (*black*) responses for older participants. (*Top right*) Increment/ON (*gray*) versus decrement/OFF (*black*) responses for younger participants. Responses to decrements are larger for decrements in both groups. (*Bottom left*) Increment/ON responses for older (*gray*) and younger (*black*) participants. (*Bottom right*) Decrement/OFF responses for older (*gray*) and younger (*black*) participants. The trailing edge of the negative peak cuts off sooner for younger participants. *Error bands* plot ±1 SEM. *Color bar* indicates *P* values for the difference between the contrast polarity conditions or age conditions, starting at *P* < 0.05 in *red*. *Asterisks* indicate runs that pass the run correction criterion at *P* < 0.05.

Response amplitudes were larger for decrements than for increments in both older (Fig. 5, top left) and younger (Fig. 5, top right) groups on a within-subject permutation test. The color bar is threshold at *P* < 0.05 (red) and runs to *P* ∼ 0 (yellow). The older and younger participant waveforms differ substantially, as can be seen in Figure 5, bottom left for ON/increment responses, and in Figure 5, bottom right for OFF/decrement responses. The dominant effect seen on between-subject permutation testing is a more rapid decline of the negative peak at ∼140 ms in the younger participants.

**Figure 6.**
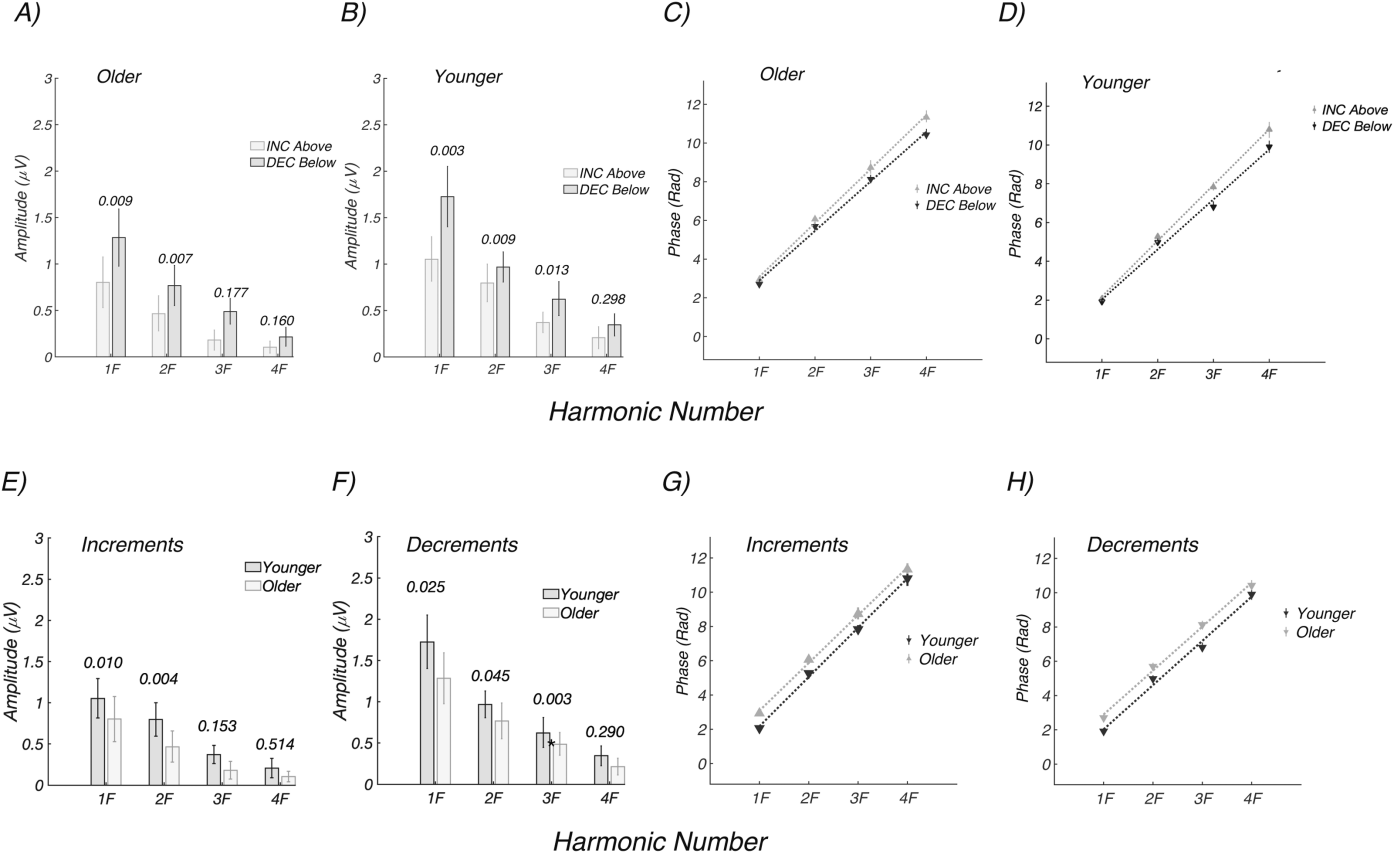
Experiment 3: Amplitude and delay estimates for older (*n* = 19) and younger (*n* = 12) participants. (A) Response amplitudes for decrements (*dark bars*) are larger than for increments (*light bars*) in the older participants. (B) Response amplitude for decrements (*dark*) are larger than for increments (*light*) in the younger participants. (C) Responses are faster for decrements than for increments in the older participants (149.3 ± 3.2 vs. 162.4 ± 2.5 ms, respectively). (D) Responses are faster for decrements than for increments in the younger participants (150.3 ± 5.0 vs. 167.7 ± 2.4 ms, respectively). (E) Response amplitudes are larger for increments in the younger participants (*dark bars*) than older participants (*light bars*). (F) Response amplitudes are larger for increments in the younger participants (*dark bars*) than older participant (*light bars*). (G) Response phase versus harmonic number functions for increments are shifted vertically for older participants. (G) Response phase versus harmonic number functions for increments are shifted vertically for older participants and have a slope difference. (H) Response phase versus harmonic number functions for decrements are shifted vertically for older participants with no change in slope.

Figure 6 plots the amplitude and phase frequency domain data for the older and younger groups. As in the time domain, the frequency domain data show larger responses for decrements than increments in both older (Fig. 6A) and younger participants (Fig. 6B). The phase of the evoked response is linear as a function of harmonic number, and the estimated delays for increment/ON and decrement/OFF responses in the older group are 162.4 ± 2.5 and 149.3 ± 3.2 ms, respectively, a difference of ∼13 ms favoring decrements (Fig. 6C). The estimated delays for increment/ON and decrement/OFF responses in the younger group are 167.7 ± 2.4 and 150.3 ± 5.0 ms, respectively, a difference of 17.4 ms favoring decrements (Fig. 6D).

Harmonic amplitudes are larger in the younger versus older participants, as shown for both increments (Fig. 6E) and decrements (Fig. 6F). In our within-group analysis of polarity-dependent delays, we focused on the slope of the phase versus harmonic number function. But when the two age groups are compared within a contrast polarity, the dominant difference is a change in the intercept rather than the slope/latency, especially for decrements (see Fig. 6G for increments and Fig. 6H for decrements). This means that there is a constant phase difference at each harmonic between the older and younger groups. This separate effect occurs in addition to the group delay difference between contrast polarity that manifests as a slope difference. Phase and group delays are typically interpreted in terms of “dispersion” in a physical medium. In neural systems, fixed delay or latency difference will create slope changes. It is likely that the constant phase differences reflect, at least in part, the relative “compactness” of the waveforms, which differ substantially between groups, as seen in Figure 5, bottom panels.

### Experiment 4: Upper versus Lower Visual Field Response Properties

The three-dimensional geometry of early retinotopic visual areas V1, V2, and V3 is such that source orientations for the upper and lower visual field representations lead to polarity reversals at the scalp and thus to the possibility of field cancellations when both upper and lower fields are stimulated at the same time.^45^^,^^46^ Moreover, previous research has found that lower field responses are typically larger than upper field responses.^47^^,^^48^ To determine the extent to which our responses were affected by field cancellation and to determine the relative magnitude of upper and lower field responses, we repeated the measurements with separate upper and lower field stimulation trials.

**Figure 7.**
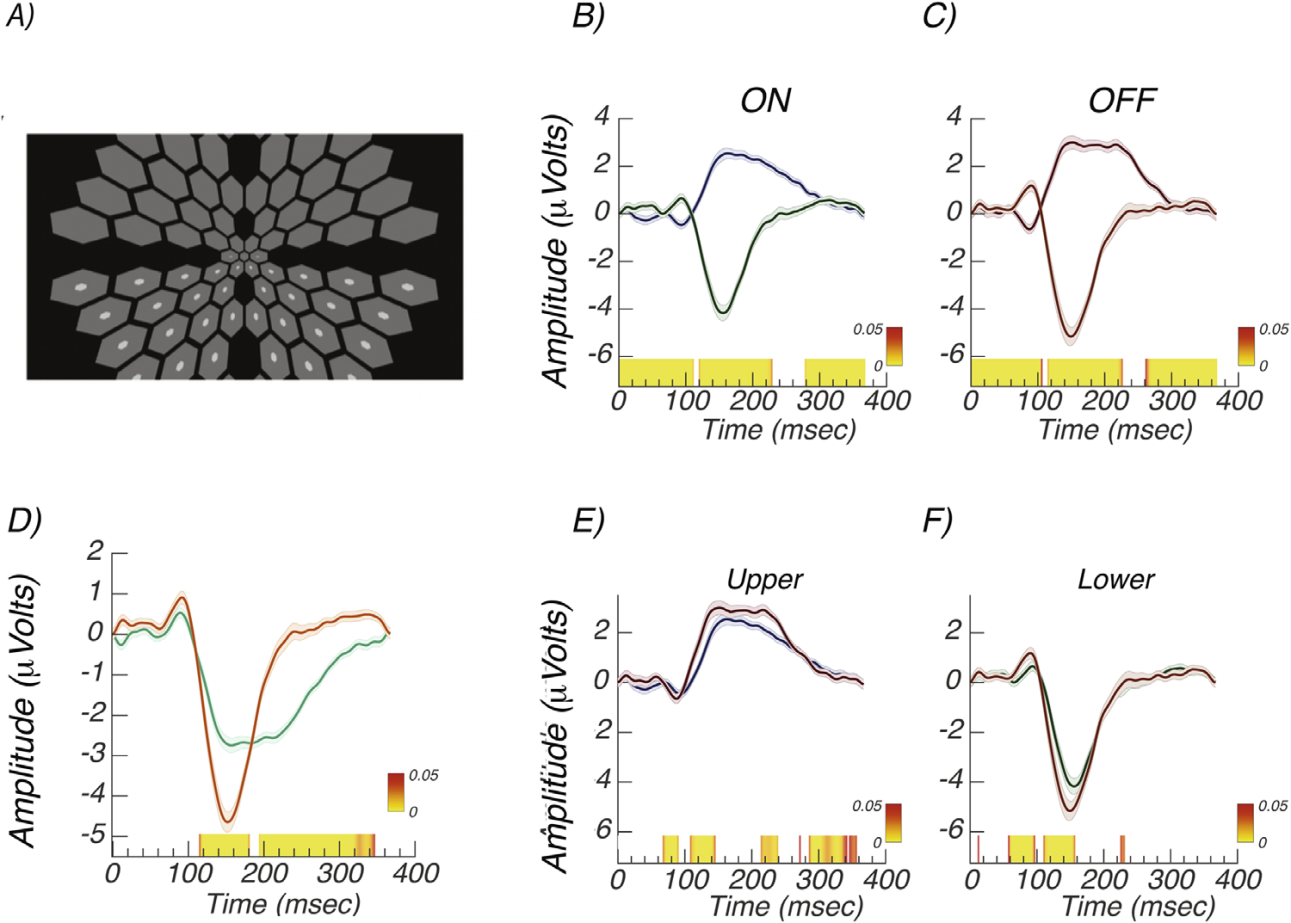
Experiment 4: Upper versus lower field responses. (A) Schematic illustration of lower field increment test configuration. The pedestals were always presented in both hemifields, but probes were presented in only one. Both increments and decrements were tested. (B) Increment/ON responses from upper (*gray*) and lower (*black*) visual fields are polarity inverted. (C) Decrement/OFF responses from upper (*gray*) and lower (*black*) visual fields are polarity inverted. (D) Comparison of upper (*green*) and lower field (*orange*) responses, pooled over contrast polarity. The polarity of the upper field responses has been inverted. (E) Comparison of increment/ON (*gray*) versus decrement/OFF (*black*) responses in the upper visual field. (F) Comparison of increment/ON (*gray*) versus decrement/OFF (*black*) responses in the lower visual field. *Error bands* are ±1 SEM.

A schematic illustration of the stimulus array used for measuring lower field increment/ON responses is shown in Figure 7A. Pedestals were presented in both hemifields, but modulating probes were presented in only one hemifield. We first consider the relative amplitude of responses from the upper and lower fields. Figure 7B shows data from the two visual fields for increment/ON responses, and Figure 7C shows the decrement/OFF responses. Notably, the response for both contrast polarities shows a sharp polarity-inverting peak at ∼90 ms for upper and lower fields that was not apparent with full-field stimulation (compare to Fig. 3A and younger participant data from Fig. 5). Additionally, the later peak at ∼150 is polarity inverted, especially on its rising phase from the two contrast polarities and inverted the polarity of the upper field RC1 component to focus the comparison on the unsigned amplitude (Fig. 7D). Lower field responses are larger by an approximate factor of 1.7 when measured between the peak at ∼90 ms and the peak at ∼150 ms.

Direct comparisons for ON/increment and OFF/decrement responses are shown in Figure 7E for the upper visual field and in Figure 7F for the lower visual field. As was seen for full-field stimulation, responses to decrements are larger and faster than for increments. The decremental response peaks occur ∼10 ms earlier than the increment responses at both early (85/90 ms) and mid-latencies (∼150 ms), and these latency differences are of the same magnitude in the upper and lower visual fields.

**Figure 8.**
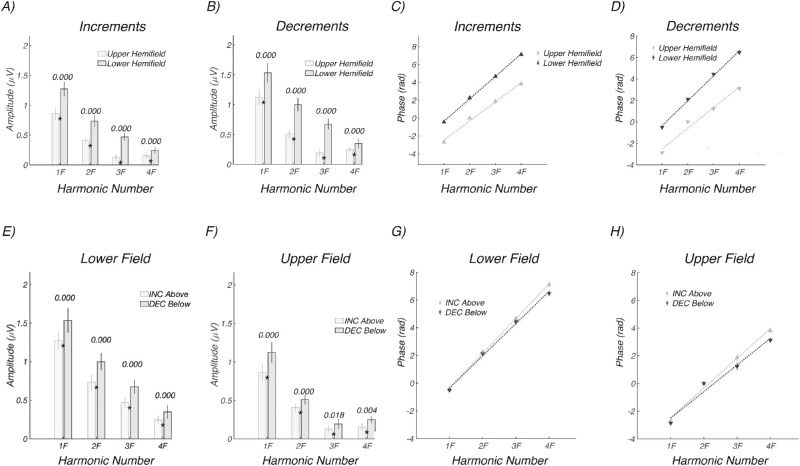
Experiment 4: Hemifield response amplitude and phase as a function of frequency (harmonic number). (A) Upper field (*light gray*) versus lower field (*dark gray*) response amplitudes for increments. (B) Upper field (*light gray*) versus lower field (*dark gray*) response amplitudes for decrements. (C, D) Corresponding delay estimates. Responses are larger and slower for the lower visual field; see text for details. (E) Lower field responses for increments (*light gray*) and decrements (*dark gray*). (F) Upper field responses for increments (*light gray*) and decrements (*dark gray*). (G, H) Corresponding latency estimates. Responses are larger and faster for decrements in both visual hemifields. See text for details.

The time-domain amplitude, waveform polarity, and latency differences seen in Figure 7 are reflected in response amplitude versus harmonic number and in the phase versus harmonic functions shown in Figure 8. Response amplitudes are larger for the lower hemifield (dark gray) than for the upper hemifield (light gray) for both increments (Fig. 8A) and decrements (Fig. 8B). The estimated delays are shorter by ∼20 to 25 ms for the upper hemifield than the lower hemifield for both increments (124.9 ± 3.8 vs. 146.1 ± 1.3 ms) and decrements (111.8 ± 6.4 vs. 135.6 ± 2.6 ms). Note that the upper and lower visual field functions are vertically offset by pi radians/180 degrees, consistent with the polarity inversions seen in the time domain.

The data are replotted as direct comparisons between contrast polarities for the two hemifields in Figures 8E and 8F. Responses are consistently larger for decrements than increments in the lower (Fig. 8E) and upper fields (Fig. 8F). Estimated latencies are faster for decrements than increments in the lower (Fig. 8G: 135.6 ± 2.6 vs. 146.1 ± 1.3 ms) and upper visual fields (Fig. 8H: 111.8 ± 6.4 vs. 124.9 ± 3.8 ms). The latency advantage for decrements is thus ∼10 ms in the lower visual field and ∼13 ms in the upper visual field, similar to what was seen for full-field stimulation in Figure 4 measured at the 94.5-cd/m^2^ pedestal luminance.

### Experiment 5: Temporally Symmetric Waveforms with Equal Mean Luminance

**Figure 9.**
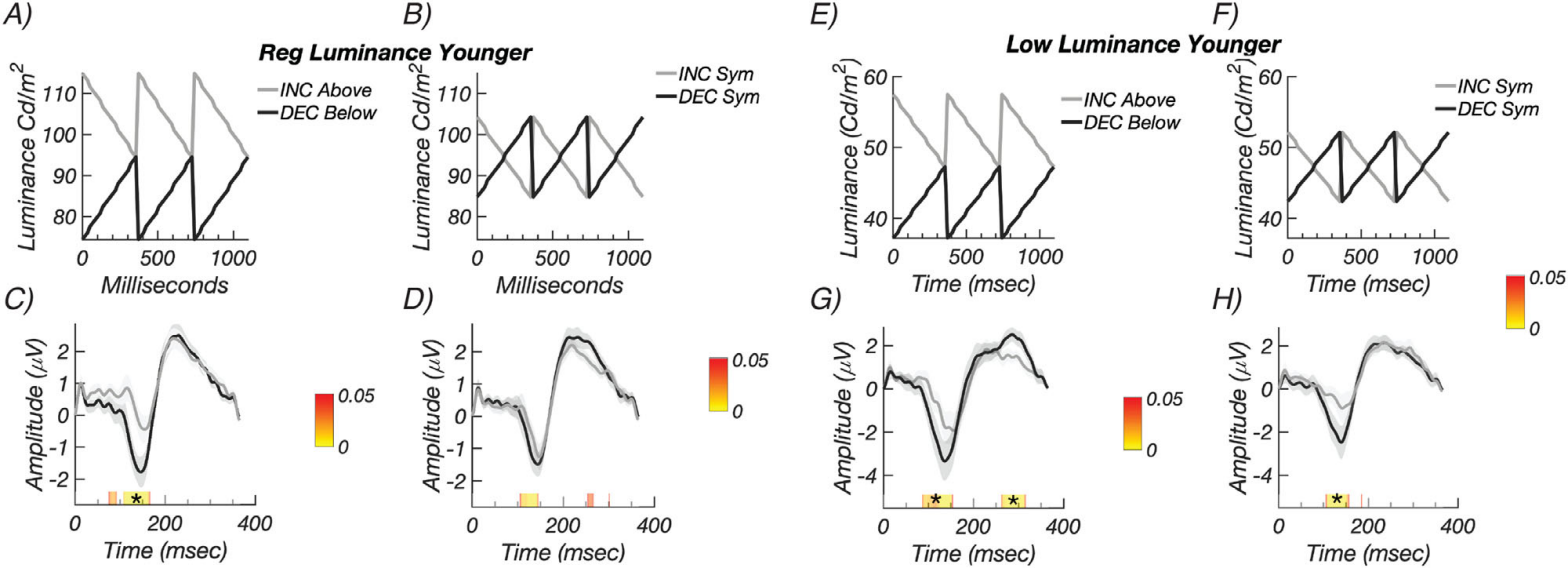
Experiment 5: Control recordings with constant Michelson contrast. (A) Schematic illustration of the biased, higher luminance decremental (*black*) and incremental (*gray*) conditions. (B) Illustration of the symmetric, equal Michelson contrast conditions using the same convention. (C) Responses for biased decremental (*black*) and incremental conditions (*gray*) are larger for decrements than increments. (D) Responses for symmetric decremental (*black*) and incremental conditions (*gray*). Response amplitudes do not differ after run correction. See text for latency estimates. The lower-luminance biased condition luminance profiles are shown schematically in panel E and the symmetric conditions in panel F. (G) Responses are larger for biased decrements than biased increments. (G) Response amplitudes are larger for symmetric decrements than for symmetric increments. See text for latency estimates. *Color bars* indicate times of significant difference (*P* < 0.05, *stars* indicate significant runs).

The use of fixed Weber contrasts in the previous experiments rests on the linearity assumption that it is only the magnitude of the probe luminance change that determines its visual effectiveness and not the light levels traversed by the probe or the relationship of the probe luminance to the pedestal luminance per se. However, if the contrast response function is nonlinear and saturating, contrast decrements may be more effective than contrast increments because they come from a less saturated portion of the contrast response function.^49^ We therefore repeated our full-field measurements in two new groups of participants, using the original “biased” increments and decrements (Figs. 9A, 9E), as well as rapid ON and rapid OFF sawtooth waveforms that modulated symmetrically about the pedestal luminance (Figs. 9B, 9F). Recordings were made in two groups of participants using pedestal luminances of 94.5 and 47.3 cd/m^2^. The symmetric modulation conditions retained the same 20% Weber contrast as used in the previous experiments, but the Michelson contrast was the now same for both contrast polarities (∼10%), instead of being ∼20% higher for the decrement/OFF stimulus relative to the increment/ON stimuli used in the previous experiments. The symmetric waveform also equated the time-averaged luminance of both probe and pedestal, equalizing their adaptation level. Symmetric sawtooth waveforms were first used in a psychophysical study^37^ that showed better sensitivity to decremental than incremental waveforms.

The results from the higher luminance recordings (*n* = 24, nine females) are shown in Figure 9C for the biased increments above the pedestal (gray) and decrements below the pedestal (black) conditions. Decremental/OFF responses were larger than incremental/ON responses. We estimated delays for each condition in this experiment from the slope of the response phase versus harmonic number plot. The corresponding delays for the data shown in Figure 9C were shorter for decremental (159.1 ± 2.8 ms) than incremental responses (178.9 ± 3.2 ms) under the biased conditions (Fig. 9C) when estimated in the frequency domain. Data from the same observers in the symmetric condition are shown in Figure 9D. Here the response amplitudes are comparable across contrast polarity, but the estimated delays were shorter for decremental (159.9 ± 3.6 ms) than incremental responses (173.0 ± 2.3 ms).

Data from the lower luminance recordings (*n* = 12, 4 female; Figs. 9E, 9F) are shown in Figure 9G for the biased conditions and in Figure 9H for the symmetric conditions. For the biased conditions shown in Figure 9G, decremental/OFF responses were larger than incremental/ON responses, and estimated delays were shorter for decremental (150.3 ± 5.0 ms) than incremental responses (167.7 ± 2.4 ms). The lower luminance symmetric conditions shown in Figure 9H have response amplitudes that were larger for decrements and estimated delays that were shorter for decremental (156.8 ± 3.3 ms) than incremental responses (173.0 ± 6.1 ms) when estimated in the frequency domain.

Responses are thus faster under equal Michelson contrast conditions under both luminance conditions but are only larger for decrements under the lower luminance conditions. Prior psychophysical work has suggested that differences between incremental and decremental sawtooth sensitivity can depend on light level, albeit over a much larger range of luminance variation.^50^

## Discussion

Here we find that SSVEPs to equal-value sawtooth increments versus decrements recorded at the scalp differ in that responses to decrements are faster and often larger than responses to increments. In addition, we found lower field responses to be substantially larger than upper field responses and that field cancellation is present with full-field displays, especially at early response latencies.

### Comparison with Previous VEP Studies of Increment and Decrement Responses

Several previous studies have compared VEPs to contrast increments and decrements. Zemon et al.^31^ used isolated checks that were sinusoidally modulated in luminance above and below the luminance of a static mid-gray background to bias responses to the ON versus OFF pathways, respectively. In the single-subject records shown in the study, responses to decrements were consistently larger than those for increments, a pattern we find to typically hold. They reported that the spatial tuning for incremental versus decremental stimuli depended on the size of the checks, being consistently larger for smaller, decremental checks than for corresponding incremental checks of the same size. Responses to our 8-arcmin probe/40-arcmin pedestal stimulus show an amplitude bias in favor of decrements under probe size conditions that produced a bias in favor of decrements in measurements by Zemon et al.^51^ (5 and 9 arcmin). A later study working within the same paradigm used sinusoidally modulated isolated checks presented at 6 and 15 Hz. Although not specifically analyzed, it is apparent from their Figure 5 that the amplitudes for increments and decrements were very similar in their normal vision control group, while we measure responses for decrements that are typically but not always larger.

The use of sinusoidal modulation biased above or below the local background level forces a difference in Michelson contrast between the two conditions. Prior authors^30^^,^^31^ replotted their data as Michelson rather than Weber contrast and found that the pattern of differences between incremental and decremental stimuli did not change. Because sawtooth stimuli are temporally asymmetric, they can be readily equated for both Michelson and Weber contrast by placing the modulation symmetrically about the local background level as we did in experiment 5. The latency advantage for decrements was still present under these conditions, and response amplitudes were larger for decrements in the lower luminance condition.

Other small studies that have compared incremental and decremental responses have found mixed results. A study using transient onset/offset of contrast increments and decrements found larger increment responses in a group of five participants.^32^ Another study used biased 2-Hz sawtooth waveforms similar to ours and found no amplitude differences between incremental versus decremental stimuli in two participants.^33^ Their use of sawtooth waveforms—as ours—was motivated by the finding that fast increment/slow decrement waveforms preferentially activated ON retinal ganglion cell in the macaque while fast decrement/slow increment waveforms activated OFF cells.^34^ The relative preference for sawtooth stimulation for preferred versus nonpreferred sawtooth stimulation in that study ranged between 3- and 10-fold below 5 Hz, suggesting a substantial but not complete ability of sawtooth stimulation to selectively stimulate ON versus OFF ganglion cells. Psychophysically, the difference in contrast sensitivity for incremental versus decremental sawtooth stimuli was on the order of 40% (e.g., a smaller difference than reported for ganglion cells).

In terms of the dynamics of ON and OFF pathways, two previous studies^31^^,^^51^ each measured the phase of the SSVEP for increments and decrements (typical response frequency of 6 Hz) and found no measurable differences for the two contrast polarities. Similarly, no latency differences were reported in the other studies that have compared incremental and decremental responses.^32^^,^^33^ In contrast to these previous studies, we find small but measurable timing differences between decremental and incremental responses. The differences are most readily apparent in the hemifield data, where we could measure both early latency and mid-latency components in the time domain and corresponding phase shifts across lower and higher harmonic responses. These differences were small, making them difficult to measure. Moreover, the use of full-field stimulation as in previous studies may complicate the measurement because responses from early visual areas may be obscured with full-field recordings (compare response amplitude at ∼90 ms in Fig. 3 to that of Fig. 7, for example).

Finally, prior work with the isolated check VEP has used rectangular arrays of small probes on a large, uniform intensity background.^52^^–^^54^ Other work on the multifocal VEP, by contrast, has scaled the elements of multielement displays in order to equate the cortical area being stimulated over retinal eccentricity.^41^ This has the benefit of roughly equating the signal-to-noise ratio for elements presented at different locations in the visual field. Here we find that scaled stimuli produce larger VEPs than the rectangular array, consistent with a role of cortical magnification in determining the amplitude of the VEP response. The magnitude of the difference between scaled and unscaled stimuli may depend on the particular size of elements used for the unscaled stimuli, a factor we did not explore.

Some of the differences between our study and these previous ones could have been due to sampling biases in small studies. Nonetheless, considering all of our data and the data from previous studies reviewed above, it appears that the relative amplitude of incremental versus decremental responses depends on the conditions of measurement and may not be a fixed property of all stimuli. Response latencies in our measurements are, by contrast, consistently faster for decrements than increments. Selective recording from separate underlying neural channels is difficult with population measures such as the SSVEP. Response magnitude measurements at the population level measured by the SSVEP depend not only on the intrinsic dynamics of the underlying populations but also their relative number. A more extensive analysis of response amplitudes at different contrast and adaptation levels would be useful to determine the generality of the effects we observe. In this respect, latency measures may be more readily interpreted in terms of cellular properties.

### Upper versus Lower Field Responses

We found that lower field responses were a factor of ∼1.7 times larger than upper field responses when averaged over contrast polarity. This hemifield asymmetry has been consistently observed in previous studies.^47^^,^^48^ Also consistent with previous work is our finding that response components invert polarity between upper and lower visual fields.^55^^–^^58^ This polarity inversion is only approximate, however. In the time domain, the shape of the upper and lower field waveform differs substantially (see Fig. 7). In the frequency domain, the upper and lower field responses differ in phase by between ∼180 degrees. This suggests the responses are dominated by early visual cortex (V1, V2, and V3), where the aggregate tissue orientation of the upper and lower visual field representations can cause scalp polarity inversions.^45^

### Comparison with Single-Unit Data

Our finding of generally larger response amplitudes for decremental sawtooth stimuli is consistent with the OFF-cell bias in cortex that has been found in single-unit studies from a variety of species.^18^^–^^20^^,^^22^^,^^59^^,^^60^ Several in vivo studies have reported that OFF cells have shorter latency than ON cells in the LGN^26^^,^^61^ and in visual cortex,^27^^,^^62^^,^^63^ a pattern we have also observed. These latency differences are small, being 10 to 16 ms (Figs. 5 and 7) in our data and between 3 and 6 ms in cat LGN,^26^^,^^61^ 3 ms in cat visual cortex,^27^ and 5 ms in macaque V1 measured with voltage-sensitive dye imaging.^63^ Note that one early in vitro study,^12^ however, found the opposite. The failure of previous VEP studies to detect small-magnitude latency differences may be been due to their limited statistical power or to the inherent sensitivity of the different measurement approaches.

OFF biases seen in the single-unit data have been argued to underlie the lower detection thresholds and faster reaction times that have been reported for contrast decrements.^27^ Our data lend additional support to this model. The amplitude biases we observe may arise in cortex, given that individual ON and OFF cells in the retina and LGN have not been reported to differ in their sensitivity.^34^ The latency biases we observe may arise subcortically and be passed on to cortex, where they appear as a constant temporal offset at all early and mid-latencies. Alternatively, the responses we measure from cortex could reflect the relative efficacy of the overall drive provided by the two pathways.

## Conclusion

In summary, we find that the sawtooth SSVEPs can be used to discriminate ON versus OFF visual pathway signaling, corroborating in human previously observed response biases measured in single cells in animal models. Future directions include exploring amplitude and timing relationships of the ON and OFF pathways as a function of visual field location, subject age, and presence of visual system disease given that preferential damage to the OFF pathway has been reported in animal models of glaucoma.^64^^–^^68^
